# Rat Milk and Plasma Immunological Profile throughout Lactation

**DOI:** 10.3390/nu13041257

**Published:** 2021-04-11

**Authors:** Blanca Grases-Pintó, Mar Abril-Gil, Paulina Torres-Castro, Margarida Castell, María J. Rodríguez-Lagunas, Francisco J. Pérez-Cano, Àngels Franch

**Affiliations:** 1 Physiology Section, Department of Biochemistry and Physiology, Faculty of Pharmacy and Food Science, University of Barcelona (UB), 08028 Barcelona, Spain; blancagrases@ub.edu (B.G.-P.); mariadelmar.abril@ub.edu (M.A.-G.); mtorreca29@alumnes.ub.edu (P.T.-C.); margaridacastell@ub.edu (M.C.); mjrodriguez@ub.edu (M.J.R.-L.); angelsfranch@ub.edu (À.F.); 2 Nutrition and Food Safety Research Institute (INSA·UB), 08921 Santa Coloma de Gramenet, Spain; 3 Centro de Investigación Biomédica en Red de Fisiopatología de la Obesidad y la Nutrición (CIBEROBN), Instituto de Salud Carlos III, 28029 Madrid, Spain

**Keywords:** breast milk, rat, immunoglobulins, adiponectin, leptin, FGF21, EGF, TGF-β

## Abstract

The composition of bioactive factors with immune activity in human breast milk is widely studied. However, the knowledge on rat milk immune factors during the whole lactation period is still scarce. This study aimed to analyze rat breast milk’s immunoglobulin (Ig) content and some critical adipokines and growth factors throughout the lactation period, and to assess relationships with corresponding plasma levels. During lactation, milk concentration of the transforming growth factor (TGF)-β2 and -β3 showed a punctual increase in the first week, whereas adiponectin and leptin remained stable. In the second period of lactation (d14–21), despite the increase in the milk epidermal growth factor (EGF), a decrease in fibroblast growth factor 21 (FGF21) was detected at day 21. Milk IgA concentration had a progressive increase during lactation, while no significant changes were found in IgM and IgG. Regarding plasma levels, a decrease in all studied adipokines was observed in the second period of lactation, with the exception of IgA and TGF-β1, which reached their highest values at the end of the study. A positive correlation in IgM, IgG, and adipokine concentration was detected between milk and plasma compartments. In summary, the changes in the pattern of these bioactive compounds in rat milk and plasma and their relationships during lactation are established.

## 1. Introduction

Breast milk is the most suitable food for the newborn. Although its composition contains all the necessary nutrients for the baby’s development and adapts to the baby’s nutritional requirements during lactation, it is known that breastfed infants may have some vitamin D or iron deficiencies [1,2,3,4], or even vitamin B12 in vegan mothers, which can be solved with supplementation to the baby or the lactating mother [5,6]. However, besides this high nutritional value, breast milk is a rich source of a great variety of bioactive compounds with health benefits for the baby [7]. Among these factors, it is worth emphasizing those that promote the maturation of the immune system (IS) in the newborn. Breast milk bioactive factors—such as immunoglobulins (Ig), lactoferrin, oligosaccharides, bacteria, adipokines, growth factors (GF), among others—provide passive protection to the baby and regulate the neonatal immune responses [8]. Although these breast milk bioactive compounds are well described in the human [8,9,10,11], the impact of external factors, such as the maternal diet or antibiotic consumption, on the breast milk immune composition is poorly studied. In this context, knowing the composition of the breast milk of experimental animals highly used in research, such as the rat, might be useful for studying different hypotheses that otherwise would not be possible to perform in human trials for various reasons. To date, there are few data available regarding the content in rat milk [12,13,14,15], and not even one study investigating the content’s pattern during the whole lactation period. In addition, despite the existing differences between humans and rodents, the rat is a species in which the length of gestation or suckling is shorter than in humans, making it useful in the research field for different purposes [16]. More accurate knowledge of the immunological profile of rat milk would be useful for interventional studies interested in the modulation of breast milk composition to have an impact on the offspring, to prepare artificial formula that better mimics breast milk, or just to explore its physiological role in the pups.

In humans, the IS is functionally immature at birth, which leads to high susceptibility to infections. The newborn usually requires approximately 30 days before it can autonomously produce enough Ig for its own protection [17]. Maternal IgG, but not IgA and IgM, can cross the placenta via the neonatal Fc receptor (FcRn) and provide passive protection to the fetus *in utero* and to the infant for months post-delivery [18,19]. After birth, Ig present in the maternal milk also provides passive immunity against infections in the newborn. Although IgA is the most abundant Ig in human milk, it also contains low concentrations of IgG and IgM [20]. Milk Ig derive from the systemic circulation and from the B lymphocytes in the mammary gland. Such cells come primarily from the maternal gut-associated lymphoid tissue through the enteromammary pathway [21]. Thus, when the newborn ingests breast milk, IgA—which is resistant to proteolytic enzymes—reaches its intestinal mucosa, where it accumulates and protects the infant against the pathogens that have been encountered previously by the mother [22].

In rats, the gestation period is around 21–24 days and there is poor transplacental transmission of Ig. As in humans, IgA and IgM do not cross the placenta, but IgG does, due to the FcRn present in this barrier [18,23,24]. Moreover, in rats, the first 21 days of life correspond to the lactating period of humans. The proportion of milk Ig isotypes changes according to the species, whereas in human milk the most abundant is IgA, which confers more protection to the gastrointestinal tract; in rat milk, IgG predominates, suggesting that its function is to boost the systemic immune response in the short term, specifically by its absorption in the neonatal intestine by a receptor-mediated endocytosis [24,25].

In addition to the Ig, human milk also has other bioactive compounds such as adipokines, including adiponectin, leptin, and fibroblast growth factor 21 (FGF21), which may have immunological properties [26]. Adiponectin appears to have a regulatory function and can play a critical anti-inflammatory role both down-modulating innate/inflammatory cells and cytokines as well as promoting regulatory T (Treg) cell function [27,28,29]. Leptin, in addition to its well-described metabolic function, also plays a more proinflammatory role in the regulation of the innate and adaptive IS [30,31,32,33], inhibiting Treg cells’ proliferation and activity [29,32]. With regard to FGF21, little is known about its function on the IS; there are few preclinical studies performed in suckling rats which show the potential role of FGF21 in promoting the maturation of the intestinal and systemic IS [34,35]. During lactation, although leptin and adiponectin are primarily transferred from the mother’s blood to the milk, they can also be synthesized by the mammary glands [26,36,37]. Nevertheless, the blood-milk transfer is the only route described for FGF21 [9]. The presence of adiponectin, leptin, and FGF21 receptors in the infant’s intestine would allow these adipokines to pass through the intestinal lumen into the newborn’s bloodstream [9,36,38]. Overall, these breast milk adipokines may influence the neonatal IS development. However, very little is known about their relationship with Ig concentration, either in plasma or in breast milk.

In addition to adipokines, breast milk also contains GF, such as epidermal growth factor (EGF) and transforming growth factor (TGF)-β, which can influence IS development. In human milk, EGF is one of the most abundant GF [39], but it is present in many other species in smaller quantities [40]. It was reported that milk EGF plays an important role in infant bowel development and in the prevention of necrotizing enterocolitis, especially in the first days of life [41]. There is not as much research evaluating the effects of EGF on the neonatal immune response [34,35,42]. With regard to human milk TGF-β, it has different functions in the infant’s gut, such as maintaining immune homeostasis, regulating inflammation (anti-inflammatory effect), promoting IgA production or participating in oral tolerance, and even influencing the composition of gut microbiota [43,44,45]. On the other hand, TGF-β promotes Treg cell differentiation and both Th17 and Th9 cell generation, and it controls the development and functionality of some innate cells [46,47].

Being aware of the important role of these breast milk components in early life and using a model to study their relevance and modulation by external factors, we hypothesize that the concentration of these milk components in rats may vary among the lactation period, as it does in other species, and these components could be linked by their plasma concentration. Thus, we aimed to investigate the concentration of the main Ig isotypes (IgA, IgM, and IgG) and other critical bioactive compounds with immunological properties (adiponectin, leptin, FGF21, EGF, and TGF-β1, -β2, -β3) in dams’ milk and plasma during the rat lactation period. In addition, the immune system mediators can be redundant in showing antagonism or having regulatory potential on other mediators. This is of great importance in the complex immunoactive composition of breast milk. Moreover, the natural evolution of one factor’s predominance may lead to a decrease in another with a similar function. Thus, we also aimed to explore the existing interactions among the factors. Finally, our secondary objective was to study the translational level of the results and to observe whether the concentration of these immune factors in rat milk were like those described in other species.

## 2. Materials and Methods

### 2.1. Animals

Eight G15 pregnant Wistar rats (RjHan:WI) were obtained from Janvier Labs (Le Genest-Saint-Isle, France) and individually housed in cages under controlled temperature and humidity conditions in a 12:12 h light:dark cycle in the Faculty of Pharmacy and Food Science animal facility. Dams were given a commercial diet corresponding to the American Institute of Nutrition 93M formulation (Harlan Teklad, Madison, WI, USA) [48] and water *ad libitum*. They were monitored daily and allowed to deliver at term naturally. The day after birth was registered as day 1 of the lactation period (and first day of pups). Litters were reduced to nine pups per lactating dam, with free access to the nipples and rat diet. Handling and sample collection were done in the same time range to avoid the influence of biological rhythms.

The Appraising Project Office’s program from the Universidad Miguel Hernández de Elche (Alicante, Spain) was carried out to estimate sample size. Considering a type I error of 0.05 (two-sided) and assuming that there is no dropout rate, it was used to give statistically significant differences among groups.

All the experimental procedures were performed according to the World Medical Association (WMA) statement on animal use in medical research and the Institutional Guidelines for the Care and Use of Laboratory Animals by the Ethical Committee for Animal Experimentation (CEEA) of the University of Barcelona (UB). The experimental project and protocols were reviewed and approved by the CEEA/UB (project identification code: 220/15, approval date: 18 March 2015) and also by the Ministry of Agriculture, Livestock, Fisheries and, Food of the Catalonia Government (DAAM; project identification code: 8521, approval date: 20 May 2015). In addition, to maximize the quality and reliability of the current experimental approach, the Animal Research-Reporting of In Vivo Experiments (ARRIVE) recommendations were also followed.

### 2.2. Experimental Design and Sample Collection

Milk and blood samples of each dam were collected on days 3, 7, 10, 14, 17, and 21 of lactation. For milk collection, litters were separated from dams, and mothers were immediately intramuscularly anesthetized with ketamine (10 mg/100 g) (Merial Laboratories S.A., Barcelona, Spain), and after 30 min, 2 U.I. of oxytocin (Syntocinon 10 U.I./mL, Alfasigma, Bologna, Italy) were intraperitoneally administered. Five min later, milking was ongoing by gentle manual stimulation from the base to the top of the teats, and milk was collected by a test tube using a manual vacuum device (Narhinel, Novartis, Basel, Switzerland) following previous laboratory procedures [12]. The overall process lasted 1–1.5 h, and the final volume obtained was always in a 0.5–1.5 mL range interval, being lower in the first collection times and higher at the end of the suckling period. Litters were reunited with their mothers after this procedure. To avoid interference with milk fat content in the immunoassays, lactic serum was isolated. Briefly, after milk centrifugation (800× *g*, 10 min, 4 °C), the fatty layer and cellular elements were removed, and the intermediate aqueous phase (lactic serum) was collected. Blood samples were obtained from the saphenous vein in heparin tubes and then centrifuged (800× *g*, 5 min, 4 °C) to obtain plasma. All samples were immediately aliquoted and stored at −80 °C until Ig, adipokine, and GF quantification analysis was conducted. At the end of the study, animals were euthanized by humane methods [12].

### 2.3. Immunoglobulin Quantification

At days 3, 7, 10, 14, 17, and 21 of lactation, IgA, IgM, and IgG concentrations were determined in lactic serum and plasma from dams using rat IgA, IgM, or IgG ELISA quantification sets (Bethyl Laboratories, Montgomery, TX, USA), as performed in previous studies [49].

### 2.4. Adipokine and Growth Factor Determination

Adiponectin, leptin, FGF21, EGF, TGF-β1, -β2, and -β3 were also quantified in dams’ lactic serum and plasma during the lactation period. For adiponectin determination, the commercial Rat Total Adiponectin/Acrp30 Quantikine ELISA Kit was used; leptin was analyzed with the Mouse/Rat Leptin Quantikine ELISA Kit; for FGF21, the Mouse/Rat FGF21 Quantikine ELISA Kit was used; and for EGF, the EGF Rat DuoSet ELISA was applied. All the ELISA Kits were supplied by R&D Systems (Minneapolis, MN, USA) and were developed following their respective manufacturer’s instructions.

The three TGF-β isoforms (TGF-β1, -β2, and -β3), which required prior activation of the latent forms, were simultaneously quantified by using the Magnetic Bead Kit (Merck Millipore, Madrid, Spain). Samples were run in the MAGPIX instrument (Luminex Corp., Austin, TX, USA), and the results were analyzed using xPONENT software (Luminex Corp.) in the Flow Cytometry Unit of the Scientific and Technological Centers of the University of Barcelona (CCiT-UB), as in previous studies [50].

All the above quantitative determinations were performed with the following limits of detection: 4000 pg/mL for adiponectin, 22 pg/mL for leptin, 3.81 pg/mL for FGF21, 0.5 pg/mL for EGF, 9.3 pg/mL for TGF-β1, 1.7 pg/mL for TGF-β2, and 2.2 pg/mL for TGF-β3.

### 2.5. Statistical Analysis

The software IBM Statistical Package for the Social Sciences (SPSS, Version 22.0, Chicago, IL, USA) was used to perform the analysis of data. Levene’s and Shapiro–Wilk tests were carried out to evaluate the homogeneity of variance and the normal distribution of the results, respectively. To assess time-dependent variables, a repeated measures ANOVA test was performed, followed by the Bonferroni’s post hoc test. Significant differences were established at *p* < 0.05.

Spearman correlation analyses between variables’ (Ig, adipokines, and GF) values in both compartments (milk and plasma) were performed on all the sampling days, and analyses considered their Area Under the Curve (AUC) during the whole lactation period (AUC_total_, d3–21), as well as in the first (AUC_d3–10_) and in the second (AUC_d14–21_) period of lactation, in order to obtain a global view of the variable changes in each stage of the lactation period.

## 3. Results

### 3.1. Immunoglobulin Profiles and Their Correlation between Milk and Plasma

**Figure 1:**
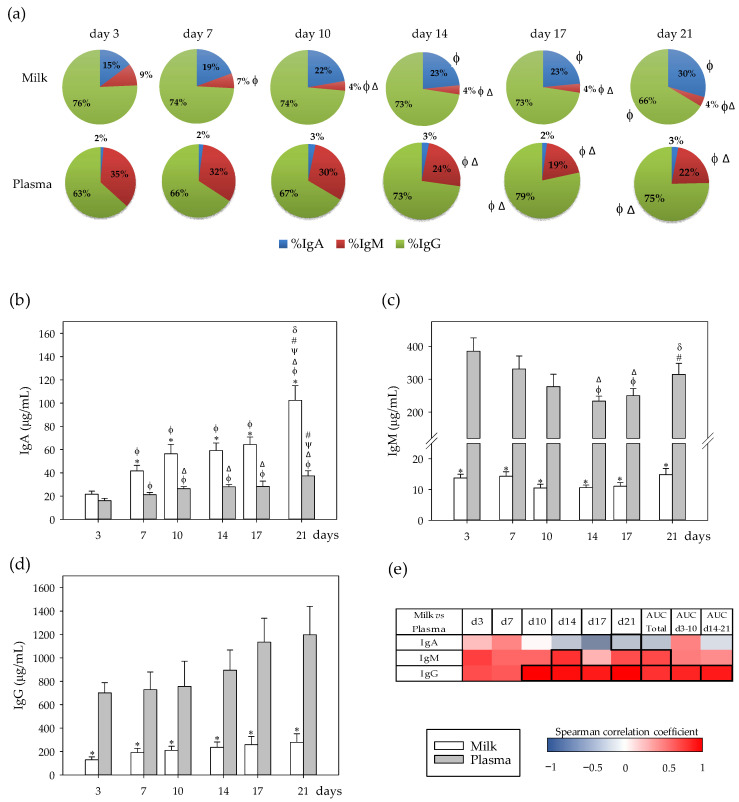
(**a**) Percentage of immunoglobulins (Ig) in milk and plasma from dams. (**b**) Milk and plasma concentration of IgA, (**c**) IgM, and (**d**) IgG throughout the lactation period. (**e**) Correlations between milk and plasma Ig levels at specific days: 3 (d3), 7 (d7), 10 (d10), 14 (d14), 17 (d17), and 21 (d21), while also considering their area under the curve during the whole lactation period (AUC_total_), and AUC on the first (AUC_d3–10_) and the second period (AUC_d14–21_) of the study. The Spearman correlation coefficient is represented in the heat map following the color in the legend. Correlations with statistical significance (*p* < 0.05) are shown in a bold frame. Statistical differences: * *p* < 0.05 vs. plasma on the same day; ^ϕ^ *p* < 0.05 vs. day 3, ^Δ^ *p* < 0.05 vs. day 7, ^ψ^ *p* < 0.05 vs. day 10, ^#^ *p* < 0.05 vs. day 14, and ^δ^ *p* < 0.05 vs. day 17 for the same type of sample (*N* = 6–8).

The concentrations of IgA, IgM, and IgG were determined in dams’ milk and plasma every 3–4 days during the lactation period, and their relative proportions were calculated (Figure 1a–d). Moreover, to ascertain the relationship between both compartments, the correlation between milk and plasma Ig levels was also analyzed (Figure 1e).

The main Ig in both the rat milk and plasma was IgG, being 63–79% of the total Ig in both compartments (Figure 1a). However, the second Ig in abundance was different depending on the compartment; in the milk it was IgA, whereas in plasma it was IgM. With regard to evolution during the lactation period, the proportion of milk IgA increased at the end of the lactation (d21), along with a decrease in IgG percentage (*p* < 0.05). The proportion of IgM in milk was higher in the first week of lactation (d3–7) than in the following days (d10–21) (*p* < 0.05). With respect to Ig present in plasma, the proportion of IgG increased in the second period of lactation (d14–21), while that of IgM decreased (*p* < 0.05), and no changes were observed in IgA (Figure 1a).

Considering the absolute plasma and milk total Ig concentrations, although the total plasma Ig amount (1000–1500 µg/mL) was not significantly modified during the lactation period, total milk Ig concentration increased during lactation, from 150–250 µg/mL in the first ten days, to 310–400 µg/mL in the second period of lactation (*p* < 0.05). Regarding particular Ig isotypes, only IgA showed changes throughout lactation in milk (*p* < 0.05), and only the IgM pattern showed differences with respect to time in plasma (*p* < 0.05). Specifically, IgA concentration was similar in both compartments at day 3 and, from that day on, IgA levels increased in both compartments (*p* < 0.05), but this increase was higher in milk, it being more abundant in this compartment than in plasma (*p* < 0.05; Figure 1b). In contrast to IgA, IgM concentration was around 25–35 times lower in milk than in plasma during lactation (*p* < 0.01; Figure 1c). Although milk IgM levels remained stable, they decreased in plasma during the first period of lactation (days 3–10) (*p* < 0.01) and increased at the end of the study (d21) (*p* < 0.05). The amount of IgG in milk was 4–6 times lower than that in the plasma compartment during the entire lactation period (Figure 1d). Although there was a tendency for IgG plasma levels to increase during the study, this increase did not reach statistical significance.

The correlations between milk and plasma concentrations were calculated and represented in a heatmap (Figure 1e). The concentration of IgA in milk inversely correlated with its plasma concentration at the end of the lactation period (*p* < 0.05). Milk IgM levels correlated positively with the AUC plasma levels throughout the lactation period, being statistically significant at day 14 and when considering the whole period as AUC_total_ (*p* < 0.05). With regard to IgG, its milk concentration was strongly linked to its plasma levels, which were statistically significant from day 10 (*p* < 0.01) and considering the AUC_total_, AUC_d3–10_ and AUC_d14–21_ (*p* < 0.05).

### 3.2. Adipokine Profiles and Their Correlation between Milk and Plasma

**Figure 2:**
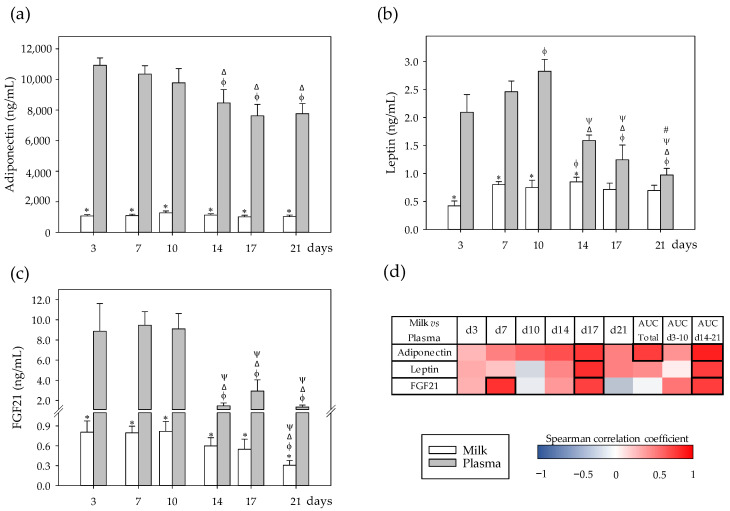
(**a**) Milk and plasma concentration of adipokines in dams: adiponectin, (**b**) leptin, and (**c**) fibroblast growth factor 21 (FGF21) throughout the lactation period. (**d**) Correlations between milk and plasma levels at specific days (3, 7, 10, 14, 17, and 21), while also considering their area under the curve during the whole lactation period (AUC_total_), and AUC on the first (AUC_d3–10_) and the second period (AUC_d14–21_) of the study. The Spearman correlation coefficient is represented in the heat map following the color in the legend. Correlations with statistical significance (*p* < 0.05) are shown in a bold frame. Statistical differences: * *p* < 0.05 vs. plasma on the same day; ^ϕ^ *p* < 0.05 vs. day 3, ^Δ^ *p* < 0.05 vs. day 7, ^ψ^ *p* < 0.05 vs. day 10, and ^#^ *p* < 0.05 vs. day 14 for the same type of sample (*N* = 6–8).

The concentration of adiponectin, leptin, and FGF21 was analyzed in milk and plasma from mothers, and the relationship between both compartments was calculated (Figure 2a–d). The three adipokines were more abundant in plasma than in milk and, in both samples, the most abundant was adiponectin, followed by FGF21 and leptin. No time-associated changes were detected in milk adiponectin levels; however, in plasma, its concentration was lower in the second period of the lactation than in the first (*p* < 0.05) (Figure 2a). The milk leptin concentration was similar during the lactation period in all days assayed, and it only exhibited a punctual increase at day 14 (*p* < 0.05) (Figure 2b), but in plasma, leptin levels increased during the first period of the lactation (d10 vs. d3) and decreased subsequently (d14–21) (*p* < 0.01). In order to understand better the relationship between adiponectin and leptin levels, their ratio was calculated. In milk, with the exception of the first collection point with very high values in the adiponectin/leptin ratio (3217.63 ± 696.66), levels were similar throughout the lactation period, being 1352.83 ± 101.89 on day 7 and 1142.58 ± 54.13 on day 21. In plasma, some changes appeared in the second period. Although both adiponectin and leptin levels decreased in that period, they did not change in the same way. Thus, the adiponectin/leptin ratio in plasma was 4642.00 ± 547.56 in the first period of lactation and reached a ratio of 6155.63 ± 696.99 in the second period (*p* < 0.05). Regarding FGF21, its concentration changed throughout lactation in both milk and plasma (*p* < 0.05). FGF21 milk concentration diminished in the second period (d14–21), being only significantly lower at day 21 compared to the previous days (*p* < 0.05) (Figure 2c). In the first period of lactation (d3–10), plasma FGF21 concentration remained stable, but it decreased markedly in the second period (d14–21) (*p* < 0.01). This pattern was similar to that observed in plasma leptin levels.

Correlation analyses revealed that the milk and plasma levels of these adipokines were, overall, positively correlated, mainly in the second period of the lactation (AUC_d14–21,_ *p* < 0.05) and particularly at day 17 (*p* < 0.05) (Figure 2d). In the case of adiponectin, this positive correlation was also observed in the AUC_total_ of the study (*p* < 0.05). FGF21 was also punctually correlated positively at day 7 between both compartments (*p* < 0.01).

### 3.3. Growth Factor Profiles and Their Correlation between Milk and Plasma

**Figure 3:**
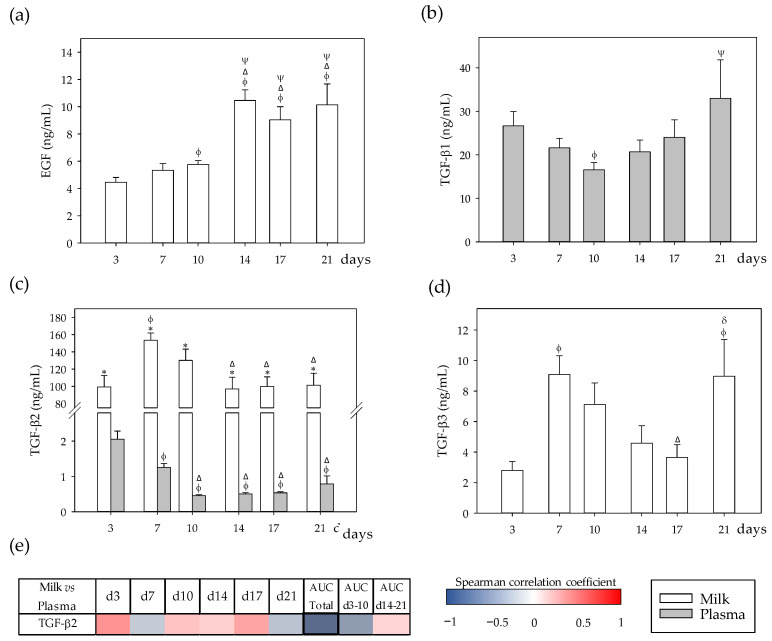
(**a**) Milk and plasma concentration of growth factors in dams: epidermal growth factor (EGF), (**b**) transforming growth factor (TGF)-β1, (**c**) TGF-β2, and (**d**) TGF-β3 throughout the lactation period. (**e**) Correlations between milk and plasma TGF-β2 levels at specific days (3, 7, 10, 14, 17, and 21), while also considering their area under the curve during the whole lactation period (AUC_total_), and AUC on the first (AUC_d3–10_) and the second period (AUC_d14–21_) of the study. The Spearman correlation coefficient is represented in the heat map following the color in the legend. Correlations with statistical significance (*p* < 0.05) are shown in a bold frame. EGF and TGF-β3 were not detected in plasma, and TGF-β1 was not detected in milk. Statistical differences: * *p* < 0.05 vs. plasma on the same day; ^ϕ^ *p* < 0.05 vs. day 3, ^Δ^ *p* < 0.05 vs. day 7, ^ψ^ *p* < 0.05 vs. day 10, and ^δ^ *p* < 0.05 vs. day 17 for the same type of sample (*N* = 6–8).

EGF and TGF-β (isoforms 1, 2, and 3) were also quantified in dams’ milk and plasma due to their role as important bioactive compounds present in milk (Figure 3a–d). All these factors, when detected, showed time-dependent changes throughout lactation in each fluid (*p* < 0.05). EGF was not detected in plasma. The concentration of milk EGF was higher in the second period of the lactation than in the first one (*p* < 0.01) (Figure 3a). With respect to TGF-β, the three isoforms showed different behaviors in milk and plasma. TGF-β1 was present in plasma but not in milk (Figure 3b), and its levels decreased in the first ten days and then increased again until the end of the lactation period (day 21, *p* < 0.05). TGF-β2 content was 50–300 times higher in milk than in plasma (Figure 3c), and its milk concentration had a maximum value at day 7 (*p* < 0.05) and decreased in the second period of the lactation (*p* < 0.05). Plasma TGF-β2 concentration diminished during the first ten days (*p* < 0.05) and remained stable in the second lactation period. Conversely, TGF-β3 was only detected in milk with maximum concentration values at days 4 and 21 (*p* < 0.05) (Figure 3d).

With regard to Spearman correlations, a negative correlation between TGF-β2 in milk and plasma was found when considering the whole lactation period (AUC_total_, *p* < 0.05).

### 3.4. Correlations between Immunoglobulins and Other Bioactive Factors in Milk and Plasma

**Figure 4:**
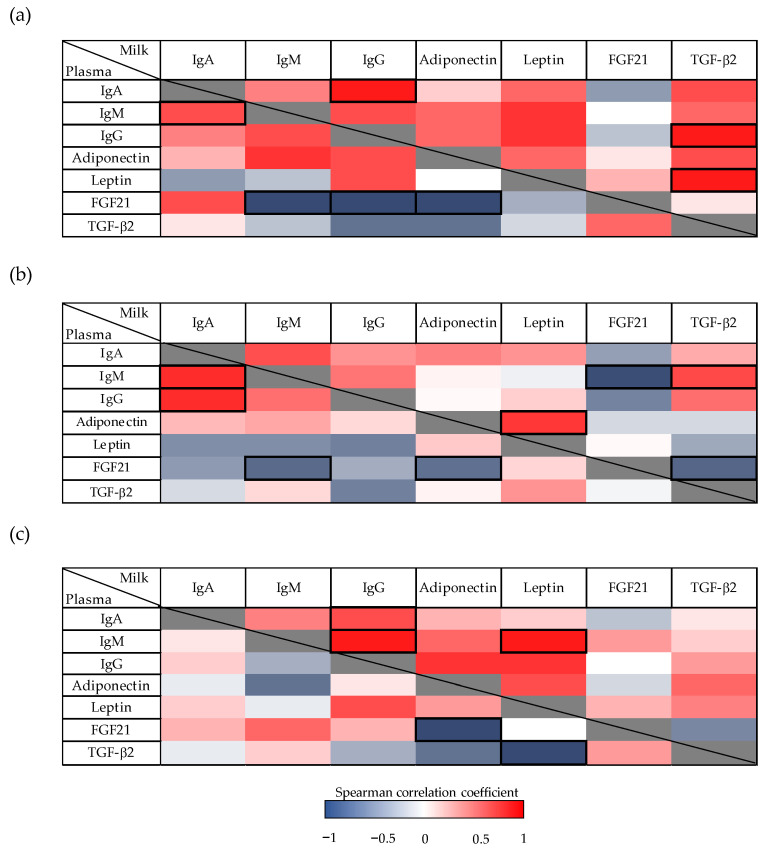
(**a**) Correlations between immunoglobulins (Ig), adipokines, and growth factor concentrations in milk and plasma from dams throughout the lactation period. Analyses were performed, considering values as their area under the curve during the whole suckling period (AUC_total_), and (**b**) AUC on the first (AUC_d3–10_) and (**c**) the second period (AUC_d14–21_) of the study. The Spearman correlation coefficient is represented in the heat map following the color in the legend. Correlations with statistical significance (*p* < 0.05) are shown in a bold frame (*N* = 6–8). TGF-β2, Transforming growth factor-β2; FGF21, fibroblast growth factor 21.

To explore whether there is a relationship between Ig, adipokines, and GF, the correlation between their contents in milk and plasma was analyzed, while considering the whole lactation period and also the first and second periods (Figure 4). When considering the whole period (AUC_total_, d3–21) (Figure 4a), positive correlations appeared between all studied components in milk, with the exception of FGF21. Milk IgG was positively correlated with TGF-β2 and plasma IgA (*p* < 0.01). Furthermore, milk TGF-β2 also showed a positive correlation with leptin (*p* < 0.01). In the plasma compartment, a positive correlation between IgA and IgM was found, whereas FGF21 was inversely correlated with IgM, IgG, and adiponectin (*p* < 0.05) (Figure 4a). These negative correlations were also observed when considering values such as the AUC_d3–10_ in the case of IgM, and the AUC_d3–10_ and AUC_d14–21_ in adiponectin (*p* < 0.05) (Figure 4b–c).

Focusing on the first period of lactation (AUC_d3–10_) (Figure 4b), IgM was negatively associated with FGF21 (*p* < 0.01) and positively correlated with TGF-β2 in milk (*p* < 0.05). Moreover, milk leptin correlated positively with adiponectin (*p* < 0.05), and FGF21 was inversely correlated with TGF-β2 (*p* < 0.05). In plasma, in addition to the negative correlation of FGF21 described previously, IgA showed a positive correlation with IgM and IgG in this first lactation period (*p* < 0.01).

Regarding the second period of lactation (AUC_d14–21_) (Figure 4c), in milk, IgG had a positive correlation with IgA and IgM (*p* < 0.05). In addition, IgM also showed a positive correlation with leptin in milk (*p* < 0.05). Finally, in the plasma compartment, a negative correlation was detected between adiponectin and FGF21 (as previously mentioned before), and between leptin and TGF-β2 (*p* < 0.05).

## 4. Discussion

The benefits of breastfeeding for the newborn are well known and include growth stimulation, enhancement of nutrient absorption, prebiotic effect, immunomodulation, and defense against pathogens, among others [22]. All these benefits are due to the magnificent composition of this biological fluid, which is well described in humans. Even though the rat is a highly-used animal species in many research works focused on the lactation period, its milk profile is not fully established. Therefore, we aimed to gain insight into rat milk composition, and in particular, we focused on the quantification of immune-active compounds such as Ig, adipokines, and GF in milk, and on the establishment of their relationship with plasma levels. It is very important to have this knowledge to incorporate these baseline measures in studies that monitor diet changes with levels of these bioactive factors in order to help with the optimization of breastmilk composition. In this line, in order to help with the design of future experiments addressing the modulation of breast milk composition and its impact on the offspring at experimental level, using the rat as a model is a suitable approach. It would help to highlight the importance of breast milk and breastfeeding practice and to implement new strategies on maternal dietary interventions, thus leading to the promotion of both mother and infant health.

Ig are bioactive factors present in breast milk that confer passive immunity to the newborn [51]. In humans, although IgG is the main Ig isotype transferred across the placenta, in milk it only represents 2% of the total Ig, with IgA being the main Ig isotype present in this fluid [19,25]. In contrast, as in bovine milk, IgG was the most abundant Ig in rat milk (about 66–76% of the entire Ig content), followed by IgA (15–30%) and finally IgM (4–9%) [52]. These results are in line with other studies that described the same pattern of Ig in rat milk [12,53], and with the fact that IgG can be absorbed in the neonatal intestine by means of the FcRn mediated endocytosis [24]. It is thought that this different Ig profile could be an adaptation to provide protection to the highly immature rat pups, due to the short developmental period *in utero* [25]. Thus, milk IgA proportion increased at the end of the lactation period, whereas that of IgG and IgM decreased, as also reported in other animals, such as pigs and horses [25].

Focusing on Ig concentrations, IgA was the only one that showed a significant change during the lactation period, increasing its concentration. In humans, although there is a huge variability among studies, the concentration of IgA is higher in colostrum (10 g/L) than in mature milk (1 g/L) [17,54]. However, studies describing Ig in rat milk are practically nonexistent, and more research is needed to elucidate their variations as well as their biological role.

IgG was the main Ig in rat plasma, being 2–3 times higher than IgM and 20–40 times higher than IgA. Although IgG predominance was maintained throughout lactation, in the second period (d14–21) an increase in IgM and a decrease in IgG percentages were observed. As in milk, little is known about the changes in the Ig profile of rat dams’ plasma during the lactation period. However, the values found from Ig in our study are similar to those described previously in rat milk and plasma at day 14 of lactation [12].

At the end of the study (day 21), milk IgA concentration was inversely correlated with its levels in plasma. This change was not observed in the first period of lactation, so the increase in milk IgA in the second period could be due mainly to filtration from the dam’s blood, rather than from local mucosal production, and therefore in parallel with a decrease in its plasma levels. On the other hand, milk IgM and IgG, above all, correlated positively with their plasma concentration, as already reported [12].

With regard to adipokines, we found that the concentration of adiponectin in rat milk was about 1300–2500 times higher than leptin during the lactation period. However, in humans, this range is much lower, being about 40 times higher than leptin [55]. On the other hand, rat milk adiponectin concentration did not vary during lactation. In the case of human milk, there is much controversy over the changes observed: While some authors describe a decrease in adiponectin content from colostrum to mature milk [55,56], others point out an increase [57,58,59]. This discrepancy could be attributed to the methodology used for milk collection, storage, or differences in the population of the study. In the present work, both adiponectin levels in milk and plasma were higher than those described in a study focused on the biological rhythms of this adipokine [15]. With regard to the concentration of leptin in milk and plasma, the values obtained were similar to those reported in that same study [15]. Moreover, we observed that plasma leptin concentration increased up to day 10 and then decreased in the subsequent days of lactation. This peak was not previously described before in dams’ plasma; however, there is a study that shows a “leptin surge”—an increase in leptin in the plasma of pups between days 7 and 10 of breastfeeding—which could be attributed to the increase in dams’ plasma and could be crucial for postnatal development [60].

In recent years, FGF21 was found in rat, mouse, and human milk, and it was shown to be relevant in the intestine of neonates [9]. In that research, its concentration in milk was higher in rodents than in humans. The levels of FGF21 in rat milk from that study were higher than the results presented here. Looking at its time course, rat milk FGF21 concentration remained stable, which partially corresponds with the results reported for FGF21 levels in human milk, which do not vary during lactation [9]. To our knowledge, this is the first time that the time course of FGF21 concentration in milk throughout the whole lactation period has been described in rats, so it needs to be further explored. On the other hand, plasma FGF21 levels found in the first period of lactation correspond with those already observed in previous studies [9].

Interestingly, for the three adipokines studied here, milk concentrations were strongly linked to their plasma levels, particularly in the second period of lactation. This positive correlation is already in rats [28,53] and also in humans [61,62,63], thereby reinforcing the connection between plasma and milk. However, other researchers also describe leptin and adiponectin mRNA transcripts in the mammary gland [64,65]. Thus, the origin of milk adiponectin and leptin could result from two pathways: one by passing from the systemic blood to the milk, and another through de novo synthesis within the mammary gland. With regard to FGF21, its passage from blood to milk and later on to rodent pups was previously demonstrated; however, its gene expression was not detected in mammary glands [9].

In the present study, EGF and TGF-β levels in rat milk through the lactation period were established. EGF is found in high concentrations in human colostrum (80 μg/L) and decreases during the lactation period (30–50 μg/L in mature milk); in addition, its levels are higher in breast milk from mothers with a very preterm delivery [54,66]. Little is known about its concentration in rat milk, and its levels in humans were determined in studies from more than 30 years ago. In rodents, the concentration of EGF in milk is about 120–450 ng/mL in mice [67,68] and 10–31 ng/mL in rat [69]. However, it should be taken into account that in this study, milk was obtained from Sprague-Dawley rats. These results are somewhat higher than those observed in the current study (4–10 ng/mL). During the lactation period, we found that EGF concentration increased in the second period of lactation, which is consistent with an increase previously reported on day 19 of lactation in rats and on day 15 in mice [67,70].

Human milk contains different isoforms of TGF-β, which are more abundant in colostrum than in mature milk [71,72], and TGF-β2 is the most abundant (95%) [72,73]. In mice, it was previously demonstrated that TGF-β1 concentration in milk is higher than TGF-β2 [68]. In our study, TGF-β1 was not detected, corresponding with Penttila et al. who did not find it in rat milk either [74]. Regarding TGF-β2, its concentration increased during the first week of the lactation period and then decreased. This result is in line with the reduction in TGF-β2 described on day 6 of lactation in rat milk [74]. With respect to TGF-β3, this is the first time that this isoform was quantified in rat breast milk. With regard to humans, in a large study including 400 gestating women from different countries, it was demonstrated that TGF-β3 was the lowest TGF-β detected, and its concentration was higher in colostrum than in mature milk [75]. This corresponds with our results, in which TGF-β3 was also found in lower concentrations than that of TGF-β2 in rat milk.

Although EGF was not detected in the rat plasma of dams during lactation, it is described in very low concentrations in humans and mice [61,76]. Thus, we cannot discard that this GF might be present in rat plasma; however, in that case it must be under the limit of detection used herein. There is not much information about the variations of plasma TGF-β isoforms during the lactation period in rat. However, in line with the results described in humans [77], we also found that plasma TGF-β1 levels were higher than those of TGF-β2.

The inverse correlation observed between TGF-β2 levels in milk and plasma could suggest that the passage of TGF-β2 from blood to milk in rats is not the main pathway as described in humans [77]. EGF was not detected in rat plasma, and the presence of EGF mRNA transcripts was described in rats’ mammary gland, thus suggesting that this gland could be the origin of milk EGF [78]. However, it is demonstrated in other species that maternal blood, rather than the mammary gland, appears to be the main source of EGF in breast milk [79].

After the establishment of rat milk and plasma concentrations of these bioactive compounds throughout the lactation period, we wondered whether they correlated among each other or with the Ig levels present in each compartment, taking into consideration that all of them are known to have immunomodulatory effects. In both milk and plasma, some Ig correlated positively. The results showed a link between them, suggesting a joint action to confer protection to the newborn. Moreover, the positive correlation described between plasma IgG and IgA matches previous findings described in mice [80]. TGF-β can increase IgA synthesis in both mice and humans, as well as IgG2b in mice [81]. Although no correlation between IgA and TGF-β was observed here, a positive link between milk IgG and TGF-β2 was detected, which could be related to the IgG2b isotype. Whereas TGF-β2 and leptin were positively correlated in milk, they were inversely correlated in rat plasma. There is little information about the relation between both bioactive compounds. However, in a study where the relationship between serum TGF-β1 and TGF-β2 and leptin was evaluated in native Chinese women, no correlation was observed between TGF-β2 and leptin [82]. With reference to the link between leptin and adiponectin in rat milk, a positive correlation between them was found in the first period of lactation. This correlation was also described by Nozhenko et al. [9] in rat milk at days 5 and 10 of lactation [9]. One of the immune functions of leptin is to enhance B-cell population by increasing proliferation and decreasing apoptotic rate [33]. Thus, this could be the reason why leptin and IgM were related in rat milk in the second period of lactation.

Finally, and as mentioned before, this study lacks data regarding the levels of these immunocomponents in pups’ plasma. However, it would be of interest to correlate all these data with the pups’ body weight and the intestinal developing aspects of the suckling rat, such as the intestinal barrier integrity and its permeability. In addition, the parallel development of the microbiota composition and the intestinal expression changes in host-bacteria interaction—through the Toll like receptors (TLR)—deserves to be studied in future studies.

## 5. Conclusions

In summary, we have established the immunological profile of Ig isotypes (IgG, IgA, and IgM), adipokines (leptin, adiponectin, and FGF21), and growth factors (EGF and TGF-β isoforms) in rat milk and plasma throughout the whole lactation period. The pattern of these bioactive compounds varied during the study, and they showed interesting correlations between their concentrations in milk and plasma, and between themselves in each compartment. Hence, this study increased the knowledge of rat breast milk composition, which is still scarce. The rat milk pattern is different from that described in humans, indicating different physiological incorporation of these bioactive factors in breast milk, as well as distinct requirements for the pups. In addition, the results described here will help with the design of further experiments addressing the modulation of breast milk composition or its impact on the offspring when using the rat as an experimental model.

## Data Availability

Not applicable.
